# Too Few Frenchmen

**Published:** 1893-01-21

**Authors:** 


### TOO FEW FRENCHMEN.

It appears that while English statesmen are bewildered by the surplus population, Frenchmen have to deplore an alarming decrease in the births, and a still more alarming increase in the deaths during the last few years. A series of accurate calculations has established the fact that such seasons of decreased population recur invariably some twenty-one years after any devastating war or pestilence, that is to say, at the period when those born in the fatal year are beginning to marry. The French are, then, at this moment feeling the recoil effect of the 1870 war. In order to recover the rate of progression of twenty-five years ago, M. de Foville has calculated that two more children must be born annually in each village throughout France. Added to this must be a corresponding decrease in infant mortality—two children less each year must die. The rate of infant mortality is indeed shamefully high, and since the births must be left to take care of themselves, attention might well be concentrated on this not unimportant point.

